# Chromatin remodelling factor BAF155 protects hepatitis B virus X protein (HBx) from ubiquitin-independent proteasomal degradation

**DOI:** 10.1080/22221751.2019.1666661

**Published:** 2019-09-19

**Authors:** Huijing Chen, Yi Zhang, Shuangshuang Ye, Qiong Wu, Youfen Lin, Kaiqin Sheng, Wannan Chen, Xinjian Lin, Xu Lin

**Affiliations:** a Key Laboratory of Gastrointestinal Cancer (Fujian Medical University), Ministry of Education, Fuzhou, People’s Republic of China; b Fujian Key Laboratory of Tumor Microbiology, Department of Medical Microbiology, Fujian Medical University, Fuzhou, People’s Republic of China

**Keywords:** Hepatitis B virus X protein, chromatin remodelling factor BAF155, ubiquitin-dependent and -independent proteasomal degradation, proteasomal subunit PSMA7, hpatocellular carcinoma

## Abstract

HBx is a short-lived protein whose rapid turnover is mainly regulated by ubiquitin-dependent proteasomal degradation pathways. Our prior work identified BAF155 to be one of the HBx binding partners. Since BAF155 has been shown to stabilize other members of the SWI/SNF chromatin remodelling complex by attenuating their proteasomal degradation, we proposed that BAF155 might also contribute to stabilizing HBx protein in a proteasome-dependent manner. Here we report that BAF155 protected hepatitis B virus X protein (HBx) from ubiquitin-independent proteasomal degradation by competing with the 20S proteasome subunit PSMA7 to bind to HBx. BAF155 was found to directly interact with HBx via binding of its SANT domain to the HBx region between amino acid residues 81 and 120. Expression of either full-length BAF155 or SANT domain increased HBx protein levels whereas siRNA-mediated knockdown of endogenous BAF155 reduced HBx protein levels. Increased HBx stability and steady-state level by BAF155 were attributable to inhibition of ubiquitin-independent and PSMA7-mediated protein degradation. Consequently, overexpression of BAF155 enhanced the transcriptional transactivation function of HBx, activated protooncogene expression and inhibited hepatoma cell clonogenicity. These results suggest that BAF155 plays important roles in ubiquitin-independent degradation of HBx, which may be related to the pathogenesis and carcinogenesis of HBV-associated HCC.

## Introduction

Hepatocellular carcinoma (HCC) is one of the most common human cancers worldwide and chronic hepatitis B virus (HBV) infection is a major risk factor in the development of HCC [1]. Among the 7 HBV viral proteins, hepatitis B virus X protein (HBx) appears to possess the most pathogenic potential as it can transactivate a variety of viral and cellular genes involved in gene transcription, intracellular signal transduction, genotoxic stress response, protein degradation, cell cycle control and apoptosis [2]. Like many other viral proteins, HBx expressed within the host cell is degraded by the proteasome pathway although the mechanisms regulating this process remain to be fully elucidated. In general, substrates of the eukaryotic 26S proteasome must be ubiquitinated in order to be recognized and subsequently degraded. The ubiquitin-proteasome pathway has been suggested to affect HBx stability as treatment with proteasome inhibitor significantly increases the steady-state level of HBx and led to the accumulation of polyubiquitinated forms [3]. However, it has also been reported that HBx may also undergo degradation through a ubiquitin-independent mechanism since HBx with all six lysines mutated showing no evidence of ubiquitination was still susceptible to proteasomal degradation [4].

SWI/SNF (Mating-type switching/Sucrose non-fermenting) is an ATP-dependent chromatin remodelling complex containing 8–10 Brg-1-associated factors (BAFs), including the core subunits of BAF155, BAF170 and SNF5 [5,6]. Aberrant expression and mutation of SWI/SNF subunits are closely associated with cancer development of many tumour types [7]. To take a relevant example, BAF155 expression is markedly increased in prostate cancer [8], breast cancer [9], cervical intraepithelial neoplasia [10] and colon cancer [11]. BAF155 expression was correlated with poor prognosis and recurrence in breast and colon cancer [9,11]. BAF155 contains several highly conserved domains such as the SANT (SWI3, ADA2, N-CoR, and TFIIIB) domain, the SWIRM (SWI3, RSC8, and MOIRA) domain and the leucine-zipper (LZ) motif although the biological function of these domains in SWI/SNF is not well understood [12]. Unlike other members of the SWI/SNF family, BAF155 does not have ATPase activity but can interact with and stabilize other members of BAFs [13]. It was observed that expressing BAF155 resulted in an increase of BAF57 [14]. The mechanism of BAF155-mediated stabilization of BAF57 involves blocking its ubiquitination by preventing interaction with thyroid hormone receptor interacting protein 12 (TRIP12), an E3 ubiquitin ligase [15]. In addition, a BAF57 mutant containing no lysine residues was found to retain its ability to be stabilized by interaction with BAF155, implying that there may also exist a ubiquitin-independent mechanism involving a direct interaction with the proteasome [15].

By use of immunoprecipitation or classical yeast two-hybrid (Y2H) screenings, a number of proteins have been identified as binding partners for HBx [16]. An early study using the yeast Y2H system identified an α subunit of the 20S proteasome, PSMA7, that could interact with HBx, and demonstrated that this interaction may be functionally relevant to the pleiotropic action of HBx [17]. Although this finding is interesting and could potentially explain the action of HBx, question remains as to whether there are other cellular factors that may regulate or interfere with the interaction of PSMA7 with HBx. In our own effort to identify HBx interacting proteins, we previously employed the CytoTrap Y2H system to screen for cellular proteins that may interact with HBx and have discovered several new candidate proteins [18]. This study further explored and confirmed one of these new candidates, BAF155, as a novel HBx-binding partner. We report here that BAF155 protects HBx from ubiquitin-independent proteasomal degradation via a previously unidentified mechanism that involves dissociation of the proteasome subunit PSMA7 from HBx thus increasing the stability and level of HBx protein as well as its pleiotropic effects.

## Materials and methods

### Cell lines and cell culture

The human hepatoma cell lines Huh7 and HepG2 as well as HepAD38 [19] and HepG2-hNTCP cells [20] were cultured in Dulbecco’s modified Eagle’s medium (Invitrogen, Carlsbad, CA, USA) at 37°C in 5% CO_2_ humidified atmosphere. The medium was supplemented with 10% fetal bovine serum. HepG2.2.15 cells harbouring four copies of HBV-DNA were cultured in modified Eagle medium (Invitrogen) with G418 (Invitrogen) at 380 μg/ml. Transfection was performed using Lipofectamine 3000 transfection reagent (Invitrogen) according to the manufacturer’s instructions.

### Production of HBV particles and infection of HepG2-hNTCP cells

HBV particles were generated from HepAD38 cells as previously described [19]. Briefly, the HepAD38 culture medium was collected and combined with 6% PEG 8000 (Sigma-Aldrich, St. Louis, MO, USA) by gentle inversion. The mixture was incubated at 4°C overnight and centrifuged at 15,000 g for 20 mins at 4°C, then the supernatant was discarded and the pellet was resuspended using DMEM in 1/100 of the original sample volume. 5 μl of the sample was used for real-time PCR to calculate the genome equivalents of HBV. HepG2-hNTCP cells that express human sodium taurocholate-cotransporting polypeptide (hNTCP) [20] were seeded in collagen-coated 24-well plates in DMEM supplemented with 10% fetal bovine serum and 1 μg/μl puromycin and 3 μg/ml doxycycline for 24 h. The cells were then infected with HBV particles at MOI (multiplicity of infection) of 100 in the presence of 4% PEG8000. After incubation for 6 days, the cells were harvested for subsequent experiments.

### Plasmids construction

pCMVTNT-BAF155 was constructed by inserting of a PCR-generated BAF155 gene (GenBank NM_003074.3) into the plasmid pCMVTNT. GST-HBx expression vector pGEX-HBx and the pRep-HBV vector (harbouring 1.2 × unit length of the HBV genome) have been previously constructed in our laboratory. pBAF155 coding for BAF155-Myc fusion protein was constructed by inserting of a PCR-generated BAF155 gene into the plasmid pcDNA3.1-Myc-His(-)A (Invitrogen). The vectors for the expression of various BAF155 deletion mutants were generated by inserting appropriate PCR fragments into vector pcDNA3.1-Myc-His(-)A. pHBx encoding HBx-Flag fusion protein was constructed by inserting of PCR-generated HBx gene fused with Flag tag sequence into plasmid pcDNA3.1-Myc-His(-)A. The vectors for the expression of various HBx deletion mutants including pHBxΔ 1–25 (deletion of amino acids 1–25), pHBxΔ 26–50 (deletion of amino acids 26–50), pHBxΔ 51–80 (deletion of amino acids 51–80), pHBxΔ81–120 (deletion of amino acids 81–120) and pHBxΔ121–154 (deletion of amino acids 121–154) fused with Flag taq sequence were generated by inserting the relative fragments that were PCR amplified from the corresponding pSos-HBx deletion mutants [18] into pcDNA3.1-Myc-His(-)A. pBAF155-SANT was constructed by insertion of PCR-generated SANT domain into pcDNA3.1-Myc-His(-)A vector. pPSMA7 encoding PSMA7-HA fusion protein was constructed by inserting of PCR-generated PSMA7 fusion HA into vector pcDNA3.1-Hygro(+) (Invitrogen). Recombinant plasmid pLL3.7-BAF155shRNA containing short hairpin RNA targeting BAF155 was constructed by GeneChem Co., Ltd. (Shanghai, China). The sequences of the paired forward and reverse primers for plasmid construction were listed in Supplementary Table S1.

### Reagents and antibodies

Cycloheximide was purchased from Cell Signaling Technology (Danvers, MA, USA). MG132 was purchased from Beyotime Biotechnology (Shanghai, China). Puromycin, doxycycline, G418 and EZview Red Anti-Flag M2 Affinity Gel was obtained from Sigma (Sigma-Aldrich). Protein A&G Agarose were obtained from Santa Cruz Biotechnology (Santa Cruz, CA, USA). Glutathione-sepharose 4B beads were from GE Healthcare (München, Germany). Anti-K48 antibody was purchased from Cell Signaling Technology detecting the endogenous levels of ubiquitination. Anti-Flag, anti-BAF155, anti-Myc, anti-GAPDH were purchased from Cell Signaling Technology. Anti-PSMA7, anti-PSMC3, anti-PSMC1, anti-PSMA1, anti-HBx were purchased from Abcam (Cambridge, UK). HRP-coupled secondary antibodies were obtained from Santa Cruz Biotechnology.

### RNA interference

For transient knockdown of PSMA7, Huh7 cells were transfected with small interfering RNA (siRNA) oligonucleotides using Lipofectamine 3000 according to the manufacturer’s instructions. The sequences of siRNA targeting PSMA7 were as follows: 5’-CCGAUGCAAGGAUAGUCAUTT-3’ and 5’-AUGACUAUCCUUGCAUCGGTT-3’ (PSMA7 siRNA1), 5’-GCGUUAUACGCAGAG CAAUTT-3’and 5’-AUUGCUCUGCGUAUAACGCTT-3’ (PSMA7 siRNA2). Recombinant plasmid pBAF155shRNA containing short hairpin RNA against BAF155 was constructed by GeneChem Co., Ltd., the target sequences were 5’-TGCAGGTCCTGTCAACTTTATTCA AGAGATAAAGTTGACAGGACCTGCTTTTTTC-3’ and 5’-TCGAGAAAAAAGCAGGTCCT GTCAACTTTATCTCTTGAATAAAGTTGACAGGACCTGCA-3’ (pBAF155shRNA1), 5’-TGC AGATGATTCATTGGATTTTCAAGAGAAATCCAATGAATCATCTGCT TTTTTC-3’ and 5’-T CGAGAAAAAAGCAGATGATTCATTGGATTTCTCTTGAAAATCCAATGAATCATCTGCA-3’ (pBAF155shRNA2). The vector of pLL3.7 expressing an shRNAi which did not target any human gene served as a control. Gene silencing effect was confirmed by western blot analysis 48 h post transfection.

### PSMA7 knockout by CRISPR/Cas9

Single guide (sg) RNAs were designed using the MIT CRISPR design software (http://crispr.mit.edu) and oligonucleotides including sgRNA sequences (PSMA7 exon 3, 5’-CAGGCCTCACCGCCGAT GCA-3′) were synthesized by Sangon Biotechnology (Shanghai, China). LentiCRISPRv2 was a gift from Brett Stringer (RRID: Addgene_98290; http://n2t.net/addgene:98290). Lentiviral vectors expressing sgRNAs targeting PSMA7 was generated as reported [21,22], verified by DNA sequencing, and used to transfect 293 T cells. The produced lentiviral particles were used to infect Huh7 cells. Stable PSMA7 knockout (Hu7-PSMA7KO) cells were selected in the presence of 1.0 μg/ml puromycin (Sigma-Aldrich) for 7 days, and the efficiency of PSMA7 knockout was validated by immunoblotting.

### GST pull-down assay

*E. coli* Rosetta (DE3) (Novagen, Madison, WI, USA) transformed with pGEX-HBx or empty vector pGEX-4T-1 was grown and induced with 0.5 mM isopropyl-β -D-thiogalactopyranoside (IPTG). The cells were harvested and disrupted by sonication in interaction buffer (phosphate buffer saline, PBS) containing 5 mM EDTA, 1 mM DTT, 1 mM PMSF and protease inhibitor cocktail (Roche Diagnostics Co., Indianapolis, IN, USA). After centrifuging, the supernatant was incubated with glutathione-sepharose 4B beads (GE Healthcare) and the GST immobilized beads were washed with interaction buffer. The purity and quantity of the bound GST and GST-HBx proteins were determined by examining SDS-PAGE gels stained with coomassie blue. TNT T7 Quick Coupled Transcription Translation System (Promega, Madison, WI, USA) was employed to express ^35^S-labeled BAF155 protein by pCMVTNT-BAF155 according to the manufacturer’s instructions. For the GST-pull-down assay, translated ^35^S-labeled BAF155 was incubated with immobilized GST-HBx or GST beads overnight at 4 °C, and then the beads were washed five times with interaction buffer. The bound proteins were subjected to 12% SDS-PAGE. After drying the gel for 15 min, the presence of ^35^S-BAF155 was detected by autoradiography.

### Co-immunoprecipitation (Co-IP) assay

For the *in vivo* Co-IP experiments, transiently transfected Huh7 cells were lysed and the soluble proteins were precleared with 100 μl of 50% slurry of protein A agarose (Invitrogen). The clear lysates were then mixed with 4 μg of antibodies and 100 μl of 50% slurry of protein A agarose. The immunoprecipitated complexes were analyzed by western blot analysis.

### Western blot analysis

Cells were lysed in western and IP cell lysis buffer (Beyotime Biotechnology, Shanghai, China) with 1 mM PMSF for 30 min at 4°C. Protein concentration was determined using the BCA protein Assay Kit (ThermoFisher Scientific, Waltham, MA, USA). The same amount of protein in each well were separated to electrophoresis in 8% or 12% SDS-PAGE gel. The proteins were transferred to PVDF membrane (GE Healthcare). The membrane was blocked 1 h with TBS-Tween 20 [50 mmol/L Tris, 160 mmol/L NaCl, 0.1% Tween 20, pH 7.8] containing 5% no-fat milk, and all subsequent steps were done in this buffer. Specific primary antibody was incubated overnight at 4°C. After intensive washing, the horseradish peroxidase-labeled HRP-coupled secondary antibody was added for 1 h and the protein was visualized with an ECL detection reagent (BeyoECL Star, Beyotime Biotechnology).

### Determination of HBx half-life

The half-life of HBx was determined as previously described with minor modification [23]. Briefly, 0.75 μg pHBx with 2.2 μg pBAF155 or 2.0 μg pBAF155 shRNA were co-transfected into Huh7 or HepG2 cells. The equal molar amounts of pcDNA3.1/Myc-His(−)A or pLL3.7 were used as a negative control. 48 h post transfection, cells were treated with 100 µg/ml of cycloheximide at indicated times. Cell were lysed with western and IP cell lysis buffer (Beyotime Biotechnology) followed by SDS-PAGE and western blot analysis.

### Ubiquitylation assay

Huh7 and HepG2 cells in 6-well plates were transfected with 0.4 µg pUb-HA coding for HA-tagged ubiquitin [23], 0.8 µg pHBx and 2.2 µg pBAF155 or pBAF155shRNA. The equal molar amounts of pcDNA3.1/Myc-His(−)A or pLL3.7 was used as a negative control. 36 h after transfection, the cells were treated with the proteasome inhibitor MG132 (20 µM, Sigma-Aldrich) for 6 h, and then harvested for the ubiquitination assay as previously reported [23].

### Colony formation assay

Huh7 and HepG2 cells in 6-well plates were transfected with empty vector or with pHBx or pBAF155 or both. 48 h after transfection, cells were cultured in geneticin selective medium for about 21 days. Surviving colonies (>50 cells per colony) were counted and photographed by a Qimaging Micropublisher 5.0 RTV microscope camera (Olympus, Tokyo, Japan).

### Cis-element luciferase reporter assay

5 × 10^5^ Huh7 cells were co-transfected with 0.75 μg of pHBx, 2.2 μg of pBAF155 and 0.4 μg each of pAP-1-luc, pNF-κB-luc, pSP-1-luc. Equal molar amounts of pcDNA3.1/Myc-His(−)A were used as a negative control. 48 h after transfection, cells were lysed and 30 μg protein were used for detection of intracellular luciferase activity (Bright-Glo Luciferase Assay System; Promega) following the manufacturer’s protocol. The light intensity was measured by a luminometer (Berthold Technologies, Oak Ridge, TN, USA). The relative luciferase unit (RLU) was obtained by comparison to the empty vector pCDNA3.1/Myc-His(−)A and was set to “1” in each experiment. Each transfection was performed in duplicate and repeated three times.

### Quantitative real-time PCR analysis

Total RNA was extracted using the TRIzol reagent (Invitrogen) and transcribed to cDNA using an ExScript RT–PCR kit (Takara, Tokyo, Japan). Quantitative real-time PCR was performed in an Mx3000P Real-Time PCR System (Agilent Technologies, Santa Clara, CA, USA) with the SYBR Premix Ex TaqTM kit (Takara) following the manufacturer’s instructions. The GAPDH gene was used as the reference gene, and relative mRNA levels were calculated using the 2^-ΔΔCt^ method. The paired forward and reverse primers were as follows: 5’-GGTGAAGACCGTGTCAGGAG-3’ and TATTCCGTTCCCTTCGGATT-3’ for c-fos, 5’-ACCACCAGCAGCGACTCTGA-3’ and 5’-TCCA GCAGAAGGTGATCCAGACT-3’ for c-myc, 5’-GTGGTTGTACGATGCATTGGTT-3’ and 5’-A TTTGTCAGCAGGACCACCA-3’ for K-ras, 5’-TGCACCACCAACTGCTTAGC-3’ and 5’-AGCTC AGGGATGACCTTGCC-3’ for GAPDH.

### Statistical analysis

Statistical analyses were performed using SPSS software (SPSS22.0). The differences between two groups were analyzed by using a Student *t* test. All values are expressed as means ± standard deviations (SD) from triplicate experiments. A *P* value of <0.05 was considered statistically significant. Experiments were performed at least three times, and representative results were shown.

## Results

### HBx interacts with BAF155

**Figure 1.**
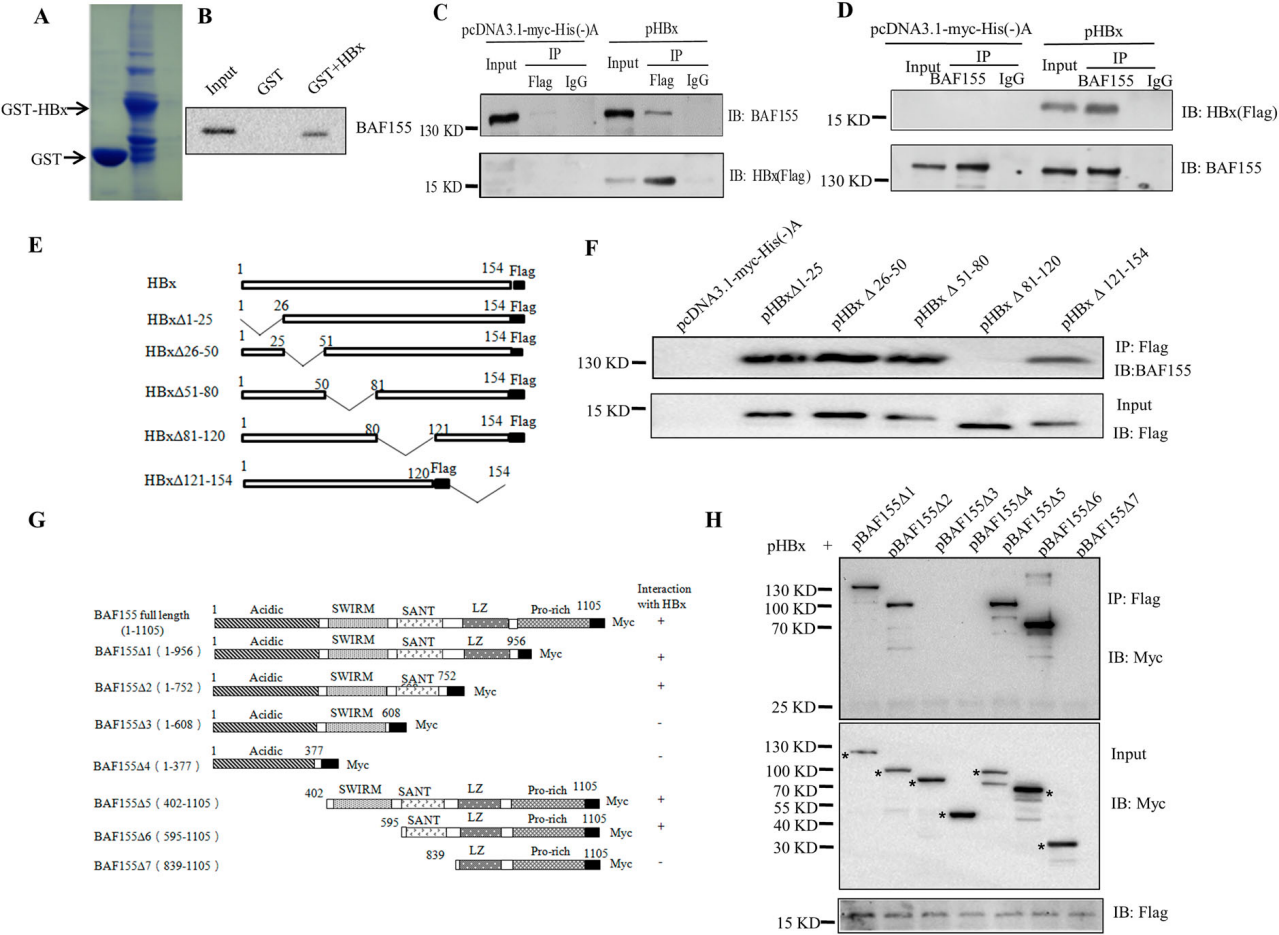
Interaction between HBx and BAF155. (**A**) Coomassie blue-stained SDS-PAGE gel showing bacterially expressed GST and GST-HBx recombinant proteins. (**B**) Representative autoradiogram of *in vitro*-translated ^35^S-BAF155 retained by GST-HBx fusion proteins from a GST pull-down assay. (**C, D**) Co-IP assay showing the interaction between HBx and endogenous BAF155 in Huh7 cells transfected with empty vector of pcDNA3.1/Myc-His(-)A or pHBx. The immunoprecipitation of BAF155 was detected for the presence of HBx and vice versa by western blot analysis. The input served as expression and specificity control for the individual proteins. (**E**) Schematic depiction of HBx and HBx mutants. The deleted region is indicated by a line with amino acid position on both ends. (**F**) Analysis of interaction between HBx mutant and BAF155 by Immunoprecipitation. (**G**) Schematic representation of wild-type BAF155 with the three major designated domains (SWIRM, SANT and LZ) and its various deletion mutants used in this study. All mutants were fused to Myc epitope at the C terminus. (**H**) Whole-cell extracts were prepared from Huh7 cells transiently transfected with pHBx and various BAF155 mutant expression vectors and immunoprecipitated with Myc antibody. The bands corresponding to the expected BAF155 protein are marked with dots. About 10% of the total input lysates used for immunoprecipitation were directly analyzed by western blotting with anti-Flag antibody (bottom panel).

To test the association between BAF155 and HBx, we first carried out a GST pull-down assay. As shown in Figure 1(A), GST and GST-HBx were well expressed in *E. coli* and immobilized on Glutathione-Sepharose beads. However, ^35^S-labeled BAF155 was retained only when reacted with bead-bound GST-HBx fusion protein not with GST alone (Figure 1B), indicating that BAF155 could interact with HBx directly*.* To confirm and extend the result of the GST pull-down assay, we performed a coimmunoprecipitation (Co-IP) study in the Huh-7 cells transiently transfected with pHBx. Figure 1(C and D) show that HBx was able to efficiently co-precipitate with endogenous BAF155, and *vice versa*. These results clearly indicate that HBx could interact with BAF155 specifically.

To determine the major domain(s) of HBx involved in the interaction with BAF155, a series of HBx deletion mutants were generated (Figure 1E) and then evaluated by Co-IP assay for their ability to bind to BAF155 (Figure 1F). Apparently, HBxΔ81-120 could not interact with BAF155, indicating that the region between HBx amino acid residues 81–120 is required for the interaction with BAF155. On the other way around, the domain(s) of BAF155 required for the interaction with HBx was also mapped. After co-transfection of Huh7 cells with different BAF155 mutant constructs with Myc tag (Figure 1G) and pHBx, whole cell extracts were immunoprecipitated with the anti-Flag antibody then the presence of BAF155 in the precipitated immunocomplexes was detected with anti-Myc antibody. As shown in Figure 1(H), BAF155Δ3 and BAF155Δ4 lacking the SANT domain could not interact with HBx, suggesting that SANT domain was indispensable for BAF155 interaction with HBx. Taken together, it is apparent that the interacting region between HBx and BAF155 resides within HBx amino acid residues 81–120 and BAF155 SANT domain.

### BAF155 positively regulates HBx protein levels

**Figure 2.**
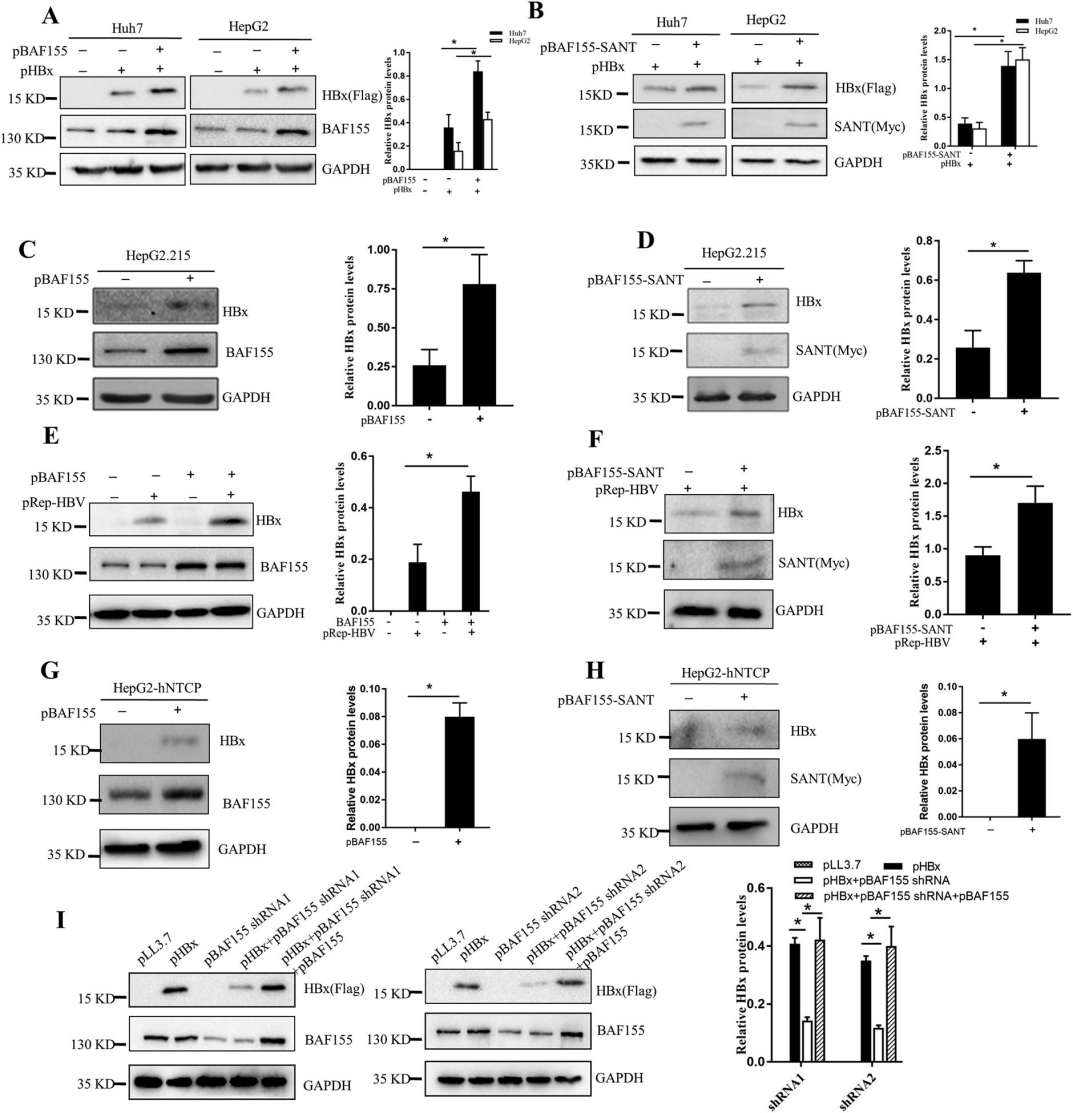
Expression of BAF155 increases HBx protein levels. (**A, B**) The protein levels of HBx in Huh7 and HepG2 cells transfected with pBAF155 (A) and pBAF155-SANT (B). (**C, D**) The protein levels of HBx in HepG2.215 cells transfected with pBAF155 (C) and pBAF155-SANT (D). (**E, F**) The protein levels of HBx in Huh7 cells co-transfected with pREP-HBV and pBAF155 or pBAF155-SANT domain. (**G, H**) The protein levels of HBx in HepG2-NTCP cells transfected with pBAF155 (G) or pBAF155-SANT domain (H). (**I**) Knockdown of endogenous BAF155 decreased HBx protein level in pHBx-transfected Huh7 cells and re-expression of BAF155 reversed the effect. pcDNA3.1/Myc-His(-)A or pLL3.7 was used as negative control. GAPDH served as a loading control. Bar graphs are the results of densitometric analysis of western blots showing the relative expression of the proteins. Values are mean ± SD, *n* = 3. *p < 0.05.

To assess the functional consequence of the interaction between HBx and BAF155, we first examined the effect of HBx on BAF155 protein levels and found that the steady-state level of BAF155 was not affected by HBx expression in both Huh7 and HepG2 cells (Supplementary Figure S1). In contrast, expression of either full-length BAF155 or its SANT domain resulted in a significant increase of HBx protein level (Figure 2A, B).

To examine whether BAF155 affected HBx expression in stable HBV-producing hepatoma cell lines HepG2.2.15 and Huh7 cells transfected with pRep-HBV harbouring 1.2 × unit length of a replication-competent wild type HBV full genome, both of which had been proved to constitutively produce infectious HBV particles [24,25], we transfected the cells with pBAF155 or pBAF155-SANT and checked for HBx expression. As expected, overexpression of full-length BAF155 or its SANT domain significantly increased HBx protein expression (Figure 2C-F). Likewise, expression of full-length BAF155 or its SANT domain in HBV-infected HepG2-NTCP cells also significantly enhanced HBx protein level (Figure 2G, H). In contrast, shRNAi-mediated knockdown of endogenous BAF155 in the pHBx-transfected Huh7 cells resulted in a significant reduction of HBx protein levels whereas re-expression of BAF155 reversed this effect by restoring HBx expression to the same level as the HBx alone-transfected cells (Figure 2I). These results suggest that BAF155 could positively regulate HBx expression.

### BAF155 sustains HBx stability

**Figure 3.**
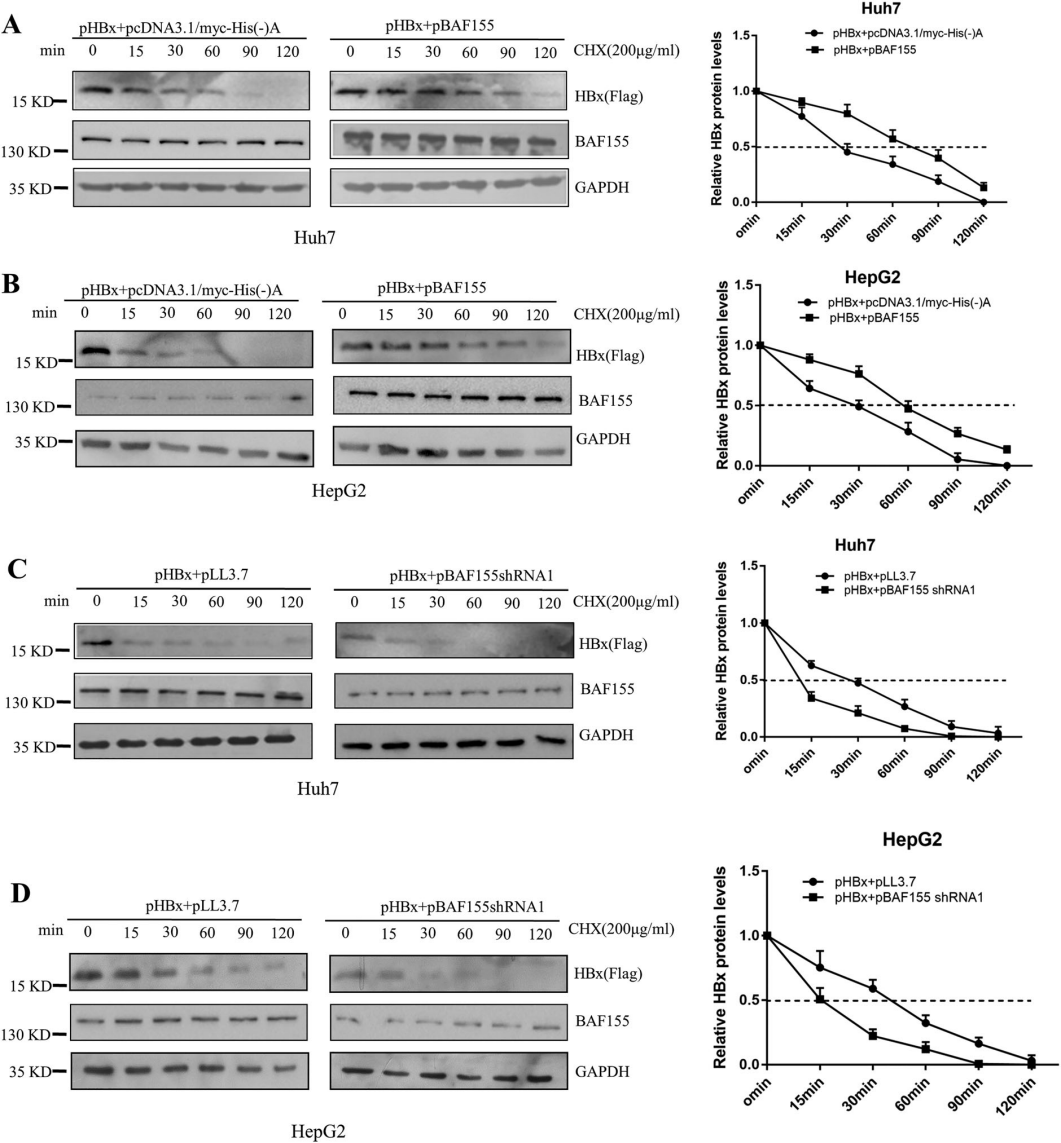
BAF155 extends HBx half-life. (**A, B**) Overexpression of BAF155 increased the HBx protein levels by extending the half-life of HBx. Huh7 (A) and HepG2 (B) cells were transfected with pHBx or in combination with pBAF155. (**C, D**) shRNAi-mediated knockdown of BAF155 reduced HBx protein levels by decreasing the half-life of HBx. Huh7 (C) and HepG2 (D) cells were transfected with pBAF155 shRNA or negative control pLL3.7. 48 h post transfection, cells were treated with 100 μg/μl cycloheximide (CHX) to inhibit de novo translation. Cells were lysed at the time points indicated.

To determine whether the increased levels of HBx by co-expression of BAF155 resulted from an extended half-life, we performed a cycloheximide chase experiment in which Huh7 and HepG2 cells were transfected with pHBx alone or in combination with pBAF155, and then continually exposed to cycloheximide for different time periods up to 120 min. Co-expression of BAF155 and HBx in Huh7 or HepG2 cells significantly extended the half-life of HBx protein from 30 to 80 min, and 30–60 min, respectively (Figure 3A, B). To examine the effect of endogenous BAF155 on the exogenously expressed HBx protein, Huh7 and HepG2 cells were co-transfected with pHBx and pBAF155 shRNA. As shown in Figure 3(C and D), knockdown of endogenous BAF155 resulted in a significant decrease in the half-life of HBx from 30 to 13 min and 30–15 min for Huh7 and HepG2 cells, respectively. These results clearly demonstrate that BAF155 increases the stability of HBx.

### BAF155 regulates HBx level via ubiquitination-independent proteasome pathway

**Figure 4.**
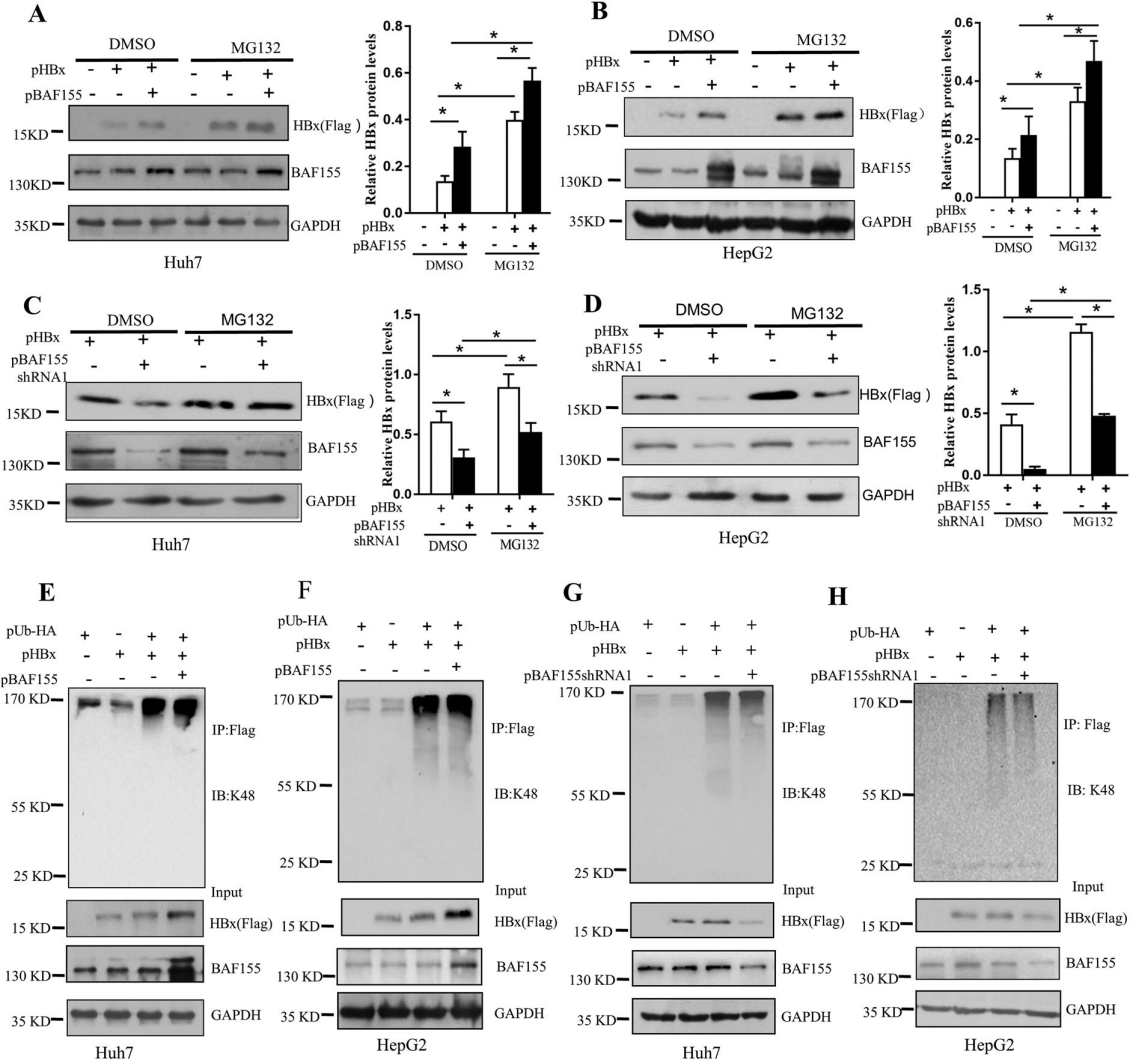
BAF155 attenuates HBx degradation in ubiquitination-independent pathway. (**A-D**) Stabilization of HBx by BAF155 through proteasomal pathway. Huh7 and HepG2 cells were co-transfected with pHBx and pBAF155 (A, B) or pBAF155-shRNA1 (C, D). 24 h after transfection, cells were treated with 20 μM MG132 for 6 h and harvested for western blot analysis. (**E-H**) BAF155 did not affect ubiquitination of HBx. Huh7 and HepG2 cells were co-transfected with pHBx and pUb-HA in combination with pBAF155 (E, F) or pBAF155-shRNA1. 24 h after transfection, cells were treated with 20 μM MG132 for 6 h. Total cell extracts were first precipitated by anti-Flag antibody and then the immunocomplex was analyzed by western blotting. Anti-K48 ubiquitin antibody was used to detect the ubiquitination level of HBx. Values are mean ± SD, *n* = 3. *p < 0.05.

To investigate whether BAF155 stabilizing effect on HBx acted through proteasome pathway, Huh7 and HepG2 cells were co-transfected with pHBx and pBAF155 or pBAF155-shRNA1 then treated with the proteasome inhibitor MG132. As shown in Figure 4(A–D), MG132 treatment further enhanced BAF155-induced protein level of HBx and rescued the reduction of HBx expression resulting from BAF155 knockdown.

To determine whether the increased level of HBx by BAF155 was due to a possible diminished ubiquitination of HBx, an *in vivo* ubiquitination assay was performed in which HBx was immunoprecipitated using anti-Flag antibody and detected by an antibody against ubiquitin. Figure 4(E–H) showed that neither overexpression of BAF155 nor knockdown of BAF155 could lead to any change of K48-linked polyubiquitination of HBx in the Huh7 and HepG2 cells, suggesting that the effect of BAF155 on stabilizing HBx does not involve induction of HBx ubiquitination.

### BAF155 stabilizes HBx through inhibition of proteasome-mediated protein degradation

**Figure 5.**
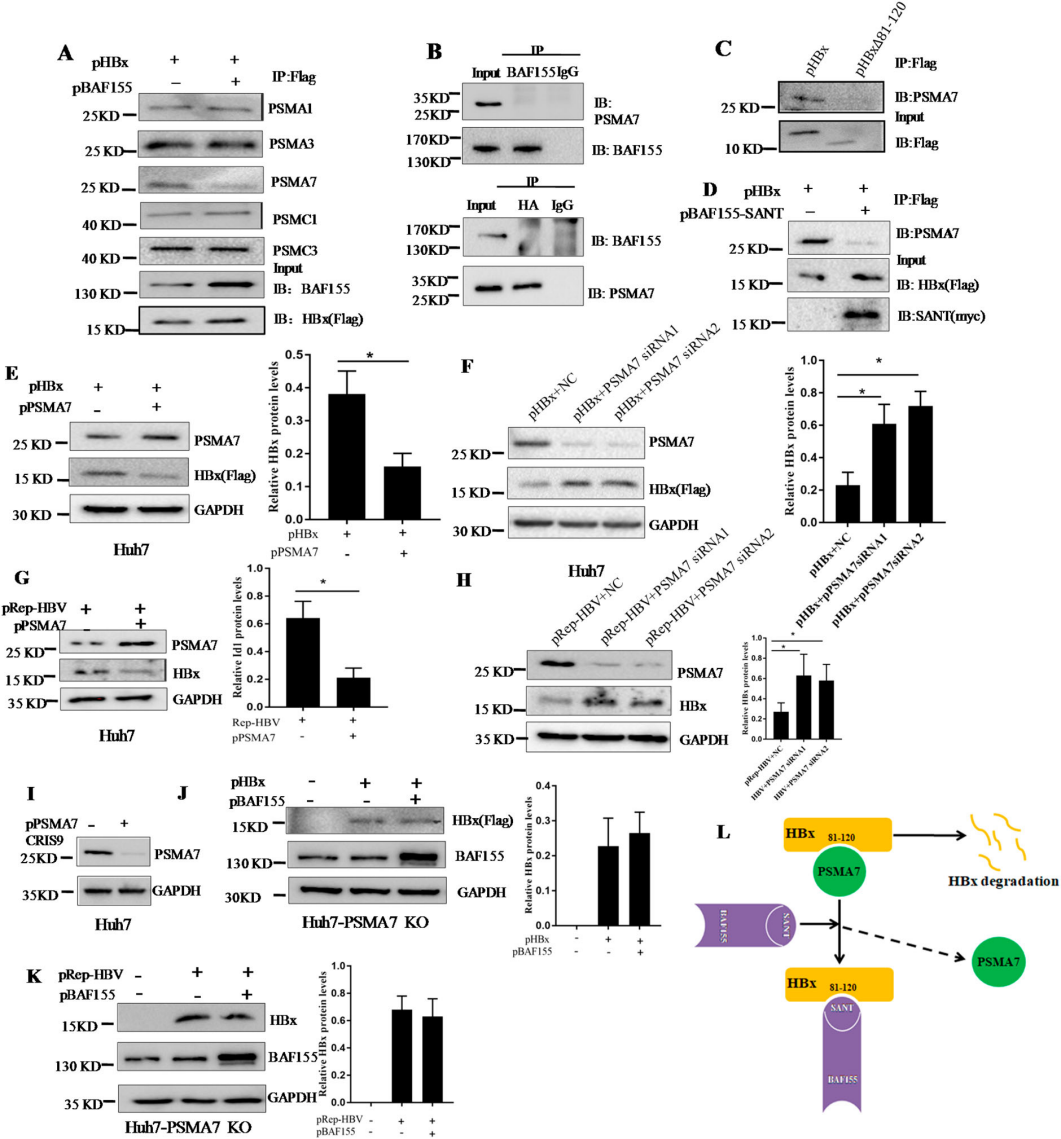
BAF155 stabilizes HBx through inhibition of proteasome-mediated protein degradation. (**A**) Coimmunoprecipitation of HBx and proteasome in the presence of proteasome inhibitor. Huh7 cells were transfected with pHBx in combination with pBAF155. 24 h after transfection, cells were treated with 20 μM MG132 for 6 h. Total cell extracts were first subjected to immunoprecipitation using anti-Flag antibody and then the immune complex was assayed by western blotting with respective antibodies. (**B**) Coimmunoprecipitation analysis of interaction between the BAF155 and PSMA7. (**C**) Coimmunoprecipitation analysis of interaction between the HBx mutant lacking aa81-120 and PSMA7. (**D**) Coimmunoprecipitation analysis of interaction between BAF155 SANT domain and PSMA7 in the presence of HBx. (**E**) Overexpression of PSMA7 decreased HBx protein levels in pHBx-transfected Huh7 cells. (**F**) Knockdown of endogenous PSMA7 increased HBx protein levels in the Huh7 cells transfected with pHBx. (**G**) Overexpression of PSMA7 decreased HBx protein levels in the Huh7 cells transfected with pRep-HBV. (**H**) Knockdown of endogenous PSMA7 increased HBx protein levels in the Huh7 cells transfected with pRep-HBV. (**I**) Knockout of PSMA7 in Huh7 cells by CRISPR/Cas9 system as examined by western blot analysis. (**J and K**) The protein levels of HBx in the PSMA7-knockout Huh7 (Huh7-PSMA7 KO) cells transfected with pBAF155 in combination with pHBx (J) or pRep-HBV (K). (**L**) Schematic models for the mechanism by which BAF155 functions to compete with PSMA7 for binding to HBx thus disrupting PSMA7 native association with HBx for HBx degradation. Values are mean ± SD, *n* = 3. *p < 0.05

Given that stabilization of HBx protein by BAF155 was not resulting from less HBx ubiquitination and that HBx has been previously reported capable of interacting with proteasome complexes such as 20S proteasome subunits PSMA1, PSMA3, PSMA7 and 19S subunits PSMC1 and PSMC3 [3,26,27], we speculated that interaction of BAF155 with HBx might competitively reduce the interaction of HBx with those proteasome complex. To test this hypothesis, the co-immunoprecipitation assay was performed in huh7 cells transfected with pHBx and pBAF155. The immunoprecipitate by anti-Flag antibody indeed contained the proteasomal subunits of PSMA1, PSMA3, PSMA7, PSMC1 and PSMC3 (Figure 5A), suggesting that HBx was actively interacting with those proteasomal subunits. Importantly, we found that overexpression of BAF155 resulted in a dramatic reduction of proteasomal subunit PSMA7 in the HBx immunocomplex while the other proteasomal subunits remained unchanged. Intriguingly, the immunoprecipitate by anti-BAF155 antibody could not detect the presence of PSMA7 and vice versa, indicating no direct interaction between BAF155 and PSMA7 (Figure 5B). These results suggest that BAF155 might block the interaction of HBx with PSMA7. Since previous mapping results revealed that the interaction of HBx and BAF155 occurred through HBx amino acid residues 81–120 and BAF155 SANT domain, we expected that HBx amino acid residues 81–120 was also essential for binding to PSMA7 and their interaction should be competed by BAF155 through the SANT domain. As predicted, HBx mutant lacking aa 81–120 was defective in binding to PSMA7 (Figure 5C) and the interaction between HBx and PSMA7 was dramatically diminished by BAF155-SANT (Figure 5D).

To verify the role of PSMA7 in HBx degradation, pHBx was co-transfected in Huh7 cells with the plasmid pPSMA7 encoding PSMA7-HA. We found that HBx protein level was significantly decreased in the co-transfected cells as compared to the cells transfected with pHBx alone (Figure 5E). Meanwhile, knockdown of endogenous PSMA7 by two specific siRNA resulted in higher HBx protein level compared with the control (Figure 1F). Similar results were obtained with the Huh7 cells transfected with pRep-HBV harbouring 1.2 × unit length of the HBV genome (Figure 5G, H). These results further support the assumption that HBx degradation is, at least in part, PSMA7 dependent.

To further confirm a central role of PSMA7 in BAF155-associated stabilization of HBx, we used the CRISPR/Cas9 system to knock out PSMA7 from Huh7 cells then the effect of BAF155 on HBx expression was examined. As shown in Figure 5(I), the sgRNA was very effective in reducing PSMA7 protein level. In the absence of PSMA7, BAF155 did lose its regulatory capability on HBx protein level (Figure 5J, K). Thus, the data is most consistent with the concept, as modelled in Figure 5(L), that BAF155 functions to compete with PSMA7 for binding to HBx thus disrupting PSMA7 native association with and degradation on HBx.

### BAF155 enhances biological functions of HBx

**Figure 6.**
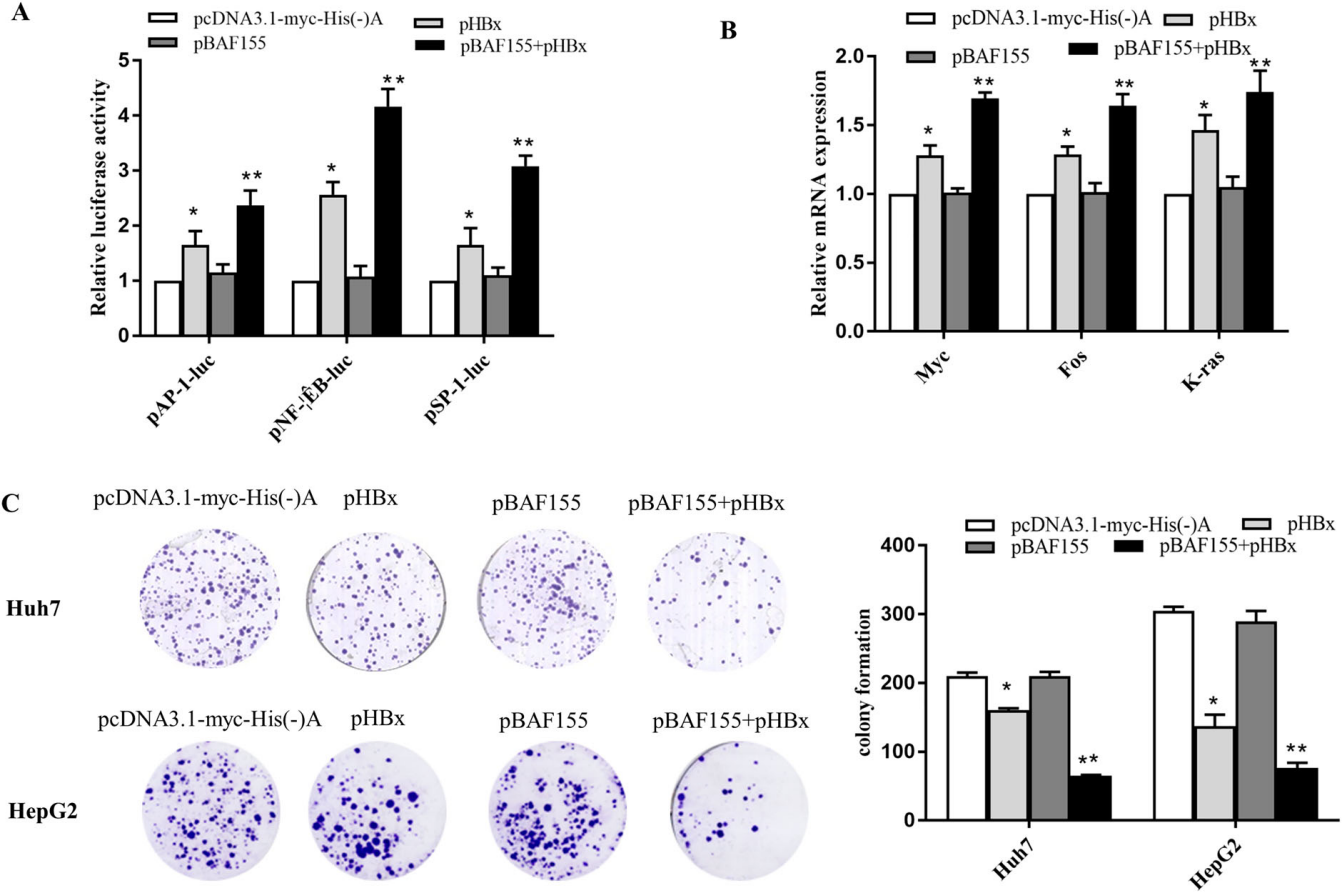
BAF155 enhances the pleiotropic function of HBx. (**A**) BAF155 augmented the transactivation activity of HBx. Huh7 cells were co-transfected with pHBx, pBAF155 and each of cis-element luciferase reporter plasmid including pGL4.1AP-1, pGL4.1NF-kB, pGL4.1SP-1. 48 h after transfection, cells were lysed and 30 μg protein was used for the detection of intracellular luciferase activity. The light intensity was measured and the relative luciferase unit (RLU) were obtained by comparison to that from the empty vector. (**B**) Quantitative PCR analysis of mRNA expression of proto-oncogenes c-myc, c-fos and K-ras in Huh7 cells co-transfected with pHBx and pBAF155. (**C**) Clonogenic survival of Huh7 and HepG2 cells transfected with pHBx or pBAF155 alone or both in the presence of G418 selection for 21 days. Values are mean ± SD, *n* = 3. *p < 0.05, **p < 0.01.

Various studies have indicated that HBx is a promiscuous transactivator and plays pivotal roles in HBV replication and hepatocarcinogenesis [28,29]. Given the observation that BAF155 is able to interact with HBx, disassociate PSMA7 from HBx and consequently increase HBx stability, we went on to explore whether the biological functions of HBx could be increased as a result of elevated HBx protein level. The luciferase reporter assay revealed that co-expression of BAF155 significantly enhanced HBx transactivation activities on AP-1, NF-κB and SP-1 whereas BAF155 overexpression alone exerted no effect on the activities of those transcriptional factors (Figure 6A). Similarly, mRNA expression of proto-oncogenes c-myc, c-fos and K-ras was also further increased in HBx and BAF155 co-transfected cells as compared to the cells expressing HBx alone. In addition, to study the functional importance of BAF155-HBx interaction we tested the clonogenicity of cells with different expression status of BAF155 and HBx by colony formation assay. As shown in Figure 6(C), BAF155 overexpression further decreased the clonogenic survival in HBx-expressing cells, which is in good agreement with the known role of HBx in inducing apoptotic cell death.

## Discussion

HBx is a rapid turnover protein having a short half-life owing to its susceptibility to proteasomal degradation [3,30]. Since a prolonged expression of this multifunctional protein is prerequisite for its pathogenicity, a specific machinery capable of regulating HBx stability must exist in the infected host cells. In the present study, we have identified the chromatin remodelling factor BAF155 as a key regulator of HBx protein levels. We demonstrate for the first time that BAF155 specifically interacts with HBx and consequently increases HBx stability and the steady-state level by inhibition of HBx ubiquitin-independent proteasomal degradation.

The physical and specific interaction between HBx and BAF155 was confirmed from a series of comprehensive binding studies. Our previous study identified BAF155 in the CytoTrap yeast two-hybrid screening system as a HBx-interacting protein. In this study we further verified the binding of HBx to BAF155 in the *in vitro* GST pull-down assay. This interaction was also corroborated *in vivo* by Co-IP studies. Using deletion analyses, it was found that HBx interacted with BAF155 at the site of contact between HBx amino acid residues 81–120 and BAF155 SANT domain. These results clearly suggest that HBx and BAF155 can interact specifically both *in vitro* and *in vivo*.

The degradation pathways of proteins mainly include lysosomal pathway, proteasome pathway and caspase pathway. Existing studies indicate that HBx can be degraded through both the ubiquitin- proteasome pathway and non-ubiquitin-dependent proteasome pathway [4]. Hu *et al* found that in the presence of the protease inhibitor MG132 or lactacystin, the half-life of HBx protein, typically 30 min, can be extended to longer than 80 min and the level of polyubiquitinated HBx was significantly increased suggesting that HBx is a typical target protein of ubiquitin-protease pathway [3]. This ubiquitin-dependent proteasomal degradation process must be highly specific in terms of requirement for the protein sequences or structural elements as the target protein has to be identified and differentiated from other proteins in the cell for degradation [31]. Accumulating evidence has indicated that the 26S proteasome and its 20S core are also involved in ubiquitin-independent protein degradation [32]. In the case of HBx, it has been demonstrated that HBx can also be degraded by a ubiquitin-independent pathway since HBx with all six of its lysines mutated, and with no evidence of ubiquitination, still serves as a proteasome substrate [4]. HBx protein was found to directly bind to various subunits of proteasome leading to non-ubiquitin-dependent rapid hydrolysis of HBx [3]. Likewise, an E3 ligase MDM2 can interact with the HBx protein and facilitates its degradation in HCC cells through a proteasome-dependent but ubiquitin-independent mechanism [33]. However, it should also be recognized that the physiological significance of the ubiquitin-dependent and -independent degradation of HBx is not clear and the relationship between its functional roles and mode of degradation is largely unknown.

BAF155 has been found to stabilize other components of SWI/SNF complexes such as BAF47, BAF60α and Brg-1 presumably by preventing them from proteasomal degradation in either ubiquitin-dependent or ubiquitin-independent manner [13,15]. However, regulatory mechanisms of BAF155 gene expression and upstream factors of BAF155 are largely unknown. Most recently, it was found that RBM15, a subunit of the m6A methyltransferase complex, interacts with BAF155 mRNA and mediates BAF155 mRNA degradation through the mRNA methylation machinery [34]. Our observation that treatment with the proteasome inhibitor MG132 enhanced BAF155-induced HBx levels or rescued the reduction of HBx due to BAF155 knockdown further supports the concept that BAF155 is important for maintaining cellular pools of HBx and that upon loss of this protection HBx is targeted for proteasomal degradation. Further *in vitro* ubiquitination experiments revealed that overexpression or knockdown of BAF155 in Huh7 and HepG2 cells did not affect the ubiquitination level of HBx, indicating that BAF155 protects HBx from degradation probably via non-ubiquitin-proteasome pathway.

Early studies documented that HBx can specifically interact with proteasome complexes such as PSMA1, PSMA7, PSMB7, PSMC1 *in vivo* and this interaction may be functionally important in the pleiotropic effect of HBx [17,26]. However, it remains controversial as to whether these interactions and HBx-mediated transactivation are due to a direct action of these ATPases on transcriptional activation or an indirect effect through the proteolytic function of the proteasome complex. We found that HBx indeed efficiently immunoprecipitated the proteasomal subunits of PSMC1, PAMA1, PSMA7, PSMA3 and PSMC3. However, when BAF155 was overexpressed, only the proteasomal subunit PSMA7 was significantly reduced in the HBx immunocomplex, implicating that BAF155 and PSMA7 might be in competition for HBx and the outcome of this competition could determine the resulting levels of HBx protein. Indeed, immunoprecipitating HBx in the presence of excessive BAF155 revealed a loss of HBx-PSMA7 interaction which strongly suggested that BAF155 interacts with and stabilizes HBx by preventing the interaction between PSMA and HBx for HBx degradation. In keeping with this, we found that knockout of PSMA7 abrogated the regulatory effect of BAF155 on HBx. Finally, we also found that the BAF155-mediated increase of HBx enhanced HBx transactivation activity, oncogene activation and proapoptotic function.

Despite the fact that various factors can downregulate or degrade HBx in cells, high-level HBx is frequently observed in HCC patients and is associated with HCC progression [35]. The mechanism by which a high-level expression of HBx is sustained in HCC remain largely unknown. Our work demonstrates that BAF155 is a novel positive regulator for HBx stability by preventing the proteasomal subunit PSMA7 from accessing its target, HBx. Furthermore, BAF155-HBx interaction appears to facilitate HBx protein to exert its pleiotropic effects. Therefore, our findings might shed new light on a novel mechanism for the elevation of HBx that is important in the pathogenesis of HBV-related hepatocellular carcinoma.

## Supplementary Material

Supplemental Material
