# Correction

**DOI:** 10.1080/22221751.2020.1755135

**Published:** 2020-05-06

**Article title:** Chromatin remodelling factor BAF155 protects hepatitis B virus X protein (HBx) from ubiquitin-independent proteasomal degradation

**Authors:** Chen, H., Zhang, Y., Ye, S., Wu, Q., Lin, Y., Shen, K., Chen, W., Lin, X., & Lin, X.

**Journal:** Emerging Microbes & Infections

**DOI:** https://doi.org/10.1080/22221751.2019.1666661

This article was originally published with an error in Figure 5. The correct version of the Figure 5 is shown below:

**Figure 5.**
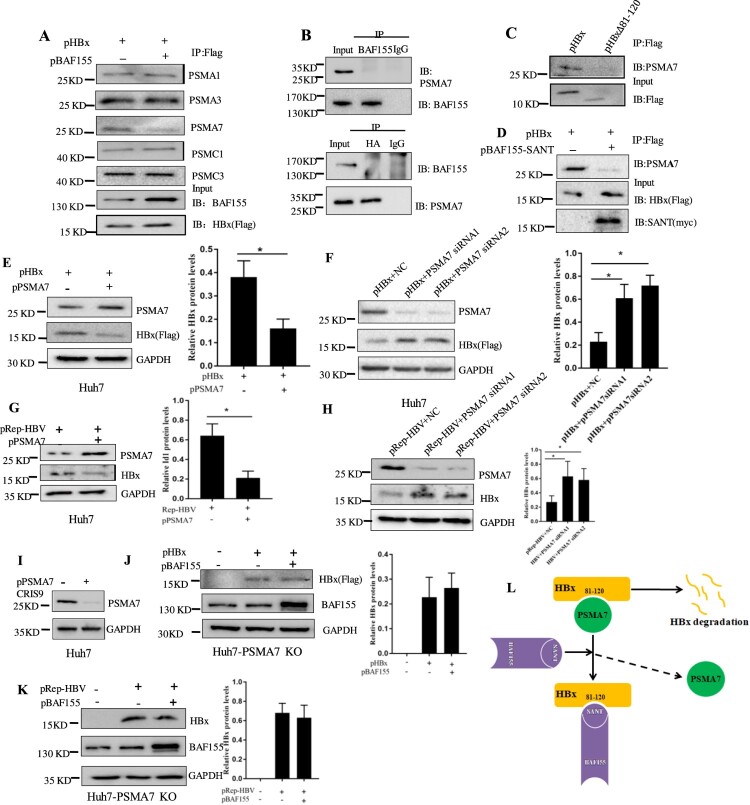
BAF155 stabilizes HBx through inhibition of proteasome-mediated protein degradation. (**A**) Coimmunoprecipitation of HBx and proteasome in the presence of proteasome inhibitor. Huh7 cells were transfected with pHBx in combination with pBAF155. 24 h after transfection, cells were treated with 20 μM MG132 for 6 h. Total cell extracts were first subjected to immunoprecipitation using anti-Flag antibody and then the immune complex was assayed by western blotting with respective antibodies. (**B**) Coimmunoprecipitation analysis of interaction between the BAF155 and PSMA7. (**C**) Coimmunoprecipitation analysis of interaction between the HBx mutant lacking aa81-120 and PSMA7. (**D**) Coimmunoprecipitation analysis of interaction between BAF155 SANT domain and PSMA7 in the presence of HBx. (**E**) Overexpression of PSMA7 decreased HBx protein levels in pHBx-transfected Huh7 cells. (**F**) Knockdown of endogenous PSMA7 increased HBx protein levels in the Huh7 cells transfected with pHBx. (**G**) Overexpression of PSMA7 decreased HBx protein levels in the Huh7 cells transfected with pRep-HBV. (**H**) Knockdown of endogenous PSMA7 increased HBx protein levels in the Huh7 cells transfected with pRep-HBV. (**I**) Knockout of PSMA7 in Huh7 cells by CRISPR/Cas9 system as examined by western blot analysis. (**J and K**) The protein levels of HBx in the PSMA7-knockout Huh7 (Huh7-PSMA7 KO) cells transfected with pBAF155 in combination with pHBx (J) or pRep-HBV (K). (**L**) Schematic models for the mechanism by which BAF155 functions to compete with PSMA7 for binding to HBx thus disrupting PSMA7 native association with HBx for HBx degradation. Values are mean ± SD, n = 3. *p < 0.05
