# Supplementary material for: Chromatin remodelling factor BAF155 protects hepatitis B virus X protein (HBx) from ubiquitin-independent proteasomal degradation
Source: Emerg Microbes Infect. 2019 Sep 19;8(1):1393–405. doi: 10.1080/22221751.2019.1666661 (PMC6758689; doi:10.1080/22221751.2019.1666661)
Supplement: Supplemental Material [file TEMI_A_1666661_SM0047.zip › Supplementary_Table_S1__9_7_19_final.docx]

**Supplementary Table S1. The sequences of the primers used in plasmid construction.**

| Plasmids | Primers |
| --- | --- |
| pCMVTNT-BAF155 | F:5’-ACGCGTCGACGCCACCATGGCCGCAGCGGCGGG-3’  R: 5’-ATAAGAATGCGGCCGCCTAAGGAGCAGCTGAGGCT-3’ |
| pBAF155 | F: 5’-CCGCTCGAGGCCACATGGCCGCAGCGGCGGGCGGC-3’  R: 5’-ATAAGAATGCGGCCGCAGGAGCAGCTGAGGCTGG-3’ |
| pBAF155∆1 | F:5’-CCGCTCGAGGCCACCATGGCCGCAGCGGCGGGCGGC-3’  R:5’-ATAAGAATGCGGCCGCCTGTCGTGCTCGTAATTC-3’ |
| pBAF155∆2 | F:5’-CCGCTCGAGGCCACCATGGCCGCAGCGGCGGGCGGC-3’  R:5’-ATAAGAATGCGGCCGCATCCACTTTCCCAGAGGC-3’ |
| pBAF155∆3 | F: 5’-CCGCTCGAGGCCACCATGGCCGCAGCGGCGGGCGGC-3’  R: 5’-ATAAGAATGCGGCCGCCTTGGAGTAAATGTCAGT-3’ |
| pBAF155∆4 | F: 5’-CCGCTCGAGGCCACCATGGCCGCAGCGGCGGGCGGC-3’  R: 5’-ATAAGAATGCGGCCGCTACAGGTGTTGGGTCTTC-3’ |
| pBAF155∆5 | F:5’-CCGCTCGAGGCCACCATGGGAGGAACTGTAGCGGAT-3’  R:5’-ATAAGAATGCGGCCGCAGGAGCAGCTGAGGCTGG-3’ |
| pBAF155∆6 | F:5’-CCGCTCGAG GCCACCATGGATTTGCAGAACTTTGGT-3’  R:5’-ATAAGAATGCGGCCGCAGGAGCAGCTGAGGCTGG-3’ |
| pBAF155∆7 | F:5’-CCGCTCGAGGCCACCATGAAAGAACTCACTGATACA-3’  R:5’-ATAAGAATGCGGCCGCAGGAGCAGCTGAGGCTGG-3’ |
| pBAF155-SANT | F:5’-CCGCTCGAGGCCACCATGAAAACATTAGCAAAGAGT-3’  R:5’-CCCAAGCTTTAGGTGAAAGGGTCTCCG-3’ |
| pPSMA7 | F: 5’-CCCAAGCTTGCCACCATGAGCTACGACCGCGCCATC-3’  R:5’-CCGCTCGAGTTACTACGAAAGAAAACAAAGTCGCATTAGACCTTGTAGCA TACCCAT-3’ |
| pHBx  pHBxΔ1–25  pHBxΔ26–50  pHBxΔ51–80  pHBxΔ81–120 | F:5’-GCTCTAGAGCCACCATGGCTGCTAGGCTG TGCT-3’  R:5-CCCAAGCTTTTACTTGTCGTCATCGTCTTTGTAGTCGGCAGAGGTGAAAAAGTTG-3’  F:5’-GCTCTAGAGCCACCATGCGCGGACGACCCCTCGCGG-3’  R:5-CCCAAGCTTTTACTTGTCGTCATCGTCTTTGTAGTCGGCAGAGGTGAAAAAGTTG-3’  F:5’-GCTCTAGAGCCACCATGGCTGCTAGGCTG TGCT-3’  R:5-CCCAAGCTT TTACTTGTCGTCATCGTCTTTGTAGTCGGCAGAGGTGAAAAAGTTG-3’  F:5’-GCTCTAGAGCCACCATGGCTGCTAGGCTGTGCT-3’  R:5-CCCAAGCTT TTACTTGTCGTCATCGTCTTTGTAGTCGGCAGAGGTGAAAAAGTTG-3’  F:5’-GCTCTAGAGCCACCATGGCTGCTAGGCTGTGCT-3’  R:5-CCCAAGCTT TTACTTGTCGTCATCGTCTTTGTAGTCGGCAGAGGTGAAAAAGTTG-3’ |
| pHBxΔ121–154 | F:5’-GCTCTAGA GCCACCATGGCTGCTAGGCTGTGCT-3’  R:5-CCCAAGCTTTTACTTGTCGTCATCGTCTTTGTAGTCCCACTCATTAAACACACAGT-3 |
