# Supplementary figures and images for: Chromatin remodelling factor BAF155 protects hepatitis B virus X protein (HBx) from ubiquitin-independent proteasomal degradation
Source: Emerg Microbes Infect. 2019 Sep 19;8(1):1393–405. doi: 10.1080/22221751.2019.1666661 (PMC6758689; doi:10.1080/22221751.2019.1666661)

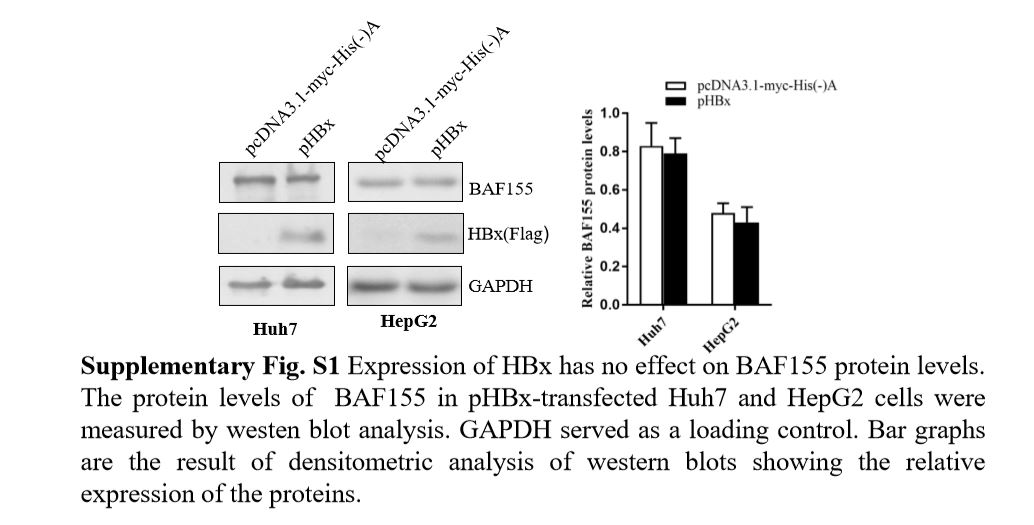

Supplement: Supplemental Material [file TEMI_A_1666661_SM0047.zip › fig.S1.JPG]
